# The Outcome of a Pregnant Woman With Tethered Cord Syndrome Following Adult Repair of Open Apina Bifida: Case Report and Literature Review

**DOI:** 10.1155/crog/6674683

**Published:** 2026-05-04

**Authors:** Jing Lyu, Huijing Zhang, Yuhua Zhao, Yunfeng Wang

**Affiliations:** ^1^ Department of Obstetrics and Gynecology, Peking University First Hospital Miyun District Hospital, Beijing, China, pkufh.com; ^2^ Department of Obstetrics and Gynecology, Peking University First Hospital, Beijing, China, pkufh.com

**Keywords:** multidisciplinary meeting, myelomeningocele, pregnancy outcome, tethered cord syndrome

## Abstract

**Background:**

Tethered cord syndrome (TCS) refers to the traction, compression, and low position of the spinal cord caused by various congenital or acquired factors. There are a few reports on pregnancy complicated by TCS.

**Case presentation:**

A 28‐year‐old Chinese pregnant nullipara woman was born with open spina bifida, who had persistently symptoms of lower extremity weakness, urinary incontinence and constipation. An elective cesarean section under general analgesia at 37 weeks of gestation after a multidisciplinary meeting and management. The pregnancy outcome was satisfactory.

**Conclusion:**

The prognosis of pregnancy complicated with TCS is good with the support of multidiscipline cooperation.

## 1. Introduction

Tethered cord syndrome (TCS) refers to the neurological impairment caused by longitudinal traction of the spinal cord, low position of the conus, and pathological changes of the terminal spinal cord. The clinical manifestations are variable, mainly including motor dysfunction, musculoskeletal deformity, neurogenic bowel and bladder, etc. [1]. In 1976, Hoffman et al. [2] first described the term TCS for nerve dysfunction caused by conus medullaris stretching. The main treatment is focusing on surgical detethering. According to Hsieh et al.’s [1] systematic review, 78%–83% of patients report significant improvement of pain, 60%–75% of patients experience stabilization of motor deficits, while 20%–30% show actual improvement in strength or gait. TCS in pregnant women is relatively rare. Robertson et al. [3] reported 68 pregnancies (50 women) complicated by spinal cord injury within 25 years. Thus, there is a scarcity of experience in prenatal and postnatal management. This case report is to share our experiencing in managing this kind of case.

## 2. Case Presentation

A 28‐year‐old Chinese nullipara woman had her routine prenatal care at Miyun District Hospital. The pregnancy was uneventful apart from a maternal complication of TCS. Since the patient suffered from urinary incontinence and difficulty in defecation. The antibiotics were prescribed to prevent urinary inflammation, and Lactulose was prescribed to relieve constipation. She was born with open spina bifida (lumbosacral myelomeningocele), resulting in severe symptoms of lower extremities weakness and difficulty in defecation and urination. She started using a wheelchair when she was 18 years old. The patient never received any treatment till she suffered from decubitus ulcers when she was 24 years old. Then, spinal cord release and myelomeningocele repair under general anesthesia were performed. However, the symptoms showed no obvious improvement. About 2 years after the surgery, the patient’s left shank was amputated due to the skin fester and loss of function.

At 34 weeks of gestation, the patient was admitted to the hospital due to “threatened preterm birth.” Since admission, steroid was prescribed to increase fetal lung maturity. Because of the patient’s medical complications, the patient was referred to the Peking University First Hospital for further management regarding timing and method of delivery.

After a multidiscipline meeting held by the Peking University First Hospital, the team decided to perform an elective cesarean section at 37 weeks of gestation, considering the increasingly deteriorating difficulties of defection and urination incontinence in the context of left lower limb amputation and restricted range of mobility. During the operation, general anesthesia and intubation were performed. A 2550 g female baby was delivered with 1 min and 5 min Apgar of 9 and 10. The patient was transferred to the surgical intensive care unit (SICU) to closely monitor her vital signs. After 5 days of expectant management, the patient was discharged without any complications.

Written informed consent was obtained for publication.

## 3. Discussion

Regarding the preconception or prenatal management, the most important thing is to recommend an enough dose of folic acid and a detailed perinatal examination to exclude neural tube defect (NTD). In our case, this patient’s TCS was caused by open spina bifida (myelomeningocele). Given that the risk for NTD in offspring of women with spinal defect is 3%–4%, compared to an approximate 0.1% in the general population [4],%, a daily supplement of 4–5 mg of folic acid was recommended [5]. Meantime, no spinal dysraphism was noted during the pregnancy or after birth.

Considering the complicating conditions, an individualized care plan for delivery should be made in conjunction with obstetricians, anesthetists, neurologists and urologists [6, 7]. The timing of delivery is a trade‐off between fetal well‐being and maternal situation. In this case, the worsening symptoms of the patient resulted in the decision to deliver the baby at 37 gestational weeks. Although Arata et al. [8] reported the possibility of successful vaginal delivery, cesarean section needs to be considered among pregnant women who are non‐ambulatory and those with fixed contractures [9–11], which occurred in this case as well.

Another issue that raised concern is the anesthetic mode. Neuraxial anesthetics are not automatically contraindicated for pregnancy with spinal dysraphism. Epidural analgesia has been reported to be successful in case series. For example, Yang and Li et al. [12] reported a successful spinal anesthesia for a pregnant woman with occult spina bifida. Todde et al. [13] reported epidural labor analgesia applied on a patient with TCS noted by MRI, but no relevant symptoms. However, they were different and less complicated compared to our case, which had open spina bifida and repaired surgery at the age of 18, though the symptoms were not relieved. Therefore, our anesthetic team chose general analgesia to avoid the operative difficulties caused by the abnormal course of the ligaments [14] and meantime to be cautious of the increased risk of perioperative respiratory problems by general analgesia [15]. The anesthetic option was an individualized approach based on the severity and complexity of the spinal dysraphism.

Overall, the prognosis of this case is positive, which is consistent with a previous study by Arata et al. [8]. The key‐point for a better prognosis is to refer those pregnant women with spina bifida to the tertiary institute, which could provide complex and individualized multi‐disciplinary management [15, 16].

## Funding

No funding was received for this manuscript.

## Disclosure

All authors have read and approved the final version of the manuscript. Yujfeng Wang has full access to all of the data in this study and takes complete responsibility for the integrity of the data and the accuracy of the data analysis. Yunfeng Wang affirms that this manuscript is an honest, accurate, and transparent account of the study being reported; that no important aspects of the study have been omitted; and that any discrepancies from the study as planned (and, if relevant, registered) have been explained.

## Conflicts of Interest

The authors declare no conflicts of interest.

## Data Availability

The data that support the findings of this study are available from the corresponding author upon reasonable request.

## References

[1] Hsieh P. , Apaydin E. , and Briggs R. G. , et al.AHRQ Comparative Effectiveness Reviews. Diagnosis and Treatment of Tethered Spinal Cord, 2024, Agency for Healthcare Research and Quality (US).

[2] Hoffman H. J. , Hendrick E. B. , and Humphreys R. P. , The Tethered Spinal Cord: Its Protean Manifestations, Diagnosis and Surgical Correction, Child’s Brain. (2004) 2, no. 3, 145–155, 10.1159/000119610, 2-s2.0-84940133732. 786565

[3] Robertson K. , Dawood R. , and Ashworth F. , Vaginal Delivery Is Safely Achieved in Pregnancies Complicated by Spinal Cord Injury: A Retrospective 25-Year Observational Study of Pregnancy Outcomes in a National Spinal Injuries Centre, BMC Pregnancy and Childbirth. (2020) 20, no. 1, 10.1186/s12884-020-2752-2. PMC6988250 31996150

[4] Berndl A. , Nosek M. , and Waddington A. , Women’s Health Guidelines for the Care of People With Spina Bifida, Journal of Pediatric Rehabilitation Medicine. (2020) 13, no. 4, 655–662, 10.3233/PRM-200757. 33325413 PMC7838966

[5] (NICE) NIfHaCE. , Evidence Reviews for High-Dose Folic Acid Supplementation Before and During the First 12 weeks of Pregnancy: Maternal and Child Nutrition: Evidence Review A, 2025, National Institute for Health and Care Excellence (NICE), (NICE Guideline, No. 247.) 2025, Available from: https://www.ncbi.nlm.nih.gov/books/NBK612331/. 39993051

[6] Crowe G. and Drew T. , Neuraxial Anaesthesia in the Parturient With Pre-Existing Structural Spinal Pathology, BJA Education. (2024) 24, no. 10, 361–370, 10.1016/j.bjae.2024.05.005. 39484011 PMC11522780

[7] Spina Bifida Association , Women’s Health Guideline for Spina Bifida, 2025, https://www.spinabifidaassociation.org/blog/womens-health-guideline/?utm_source=chatgpt.com.

[8] Arata M. , Grover S. , Dunne K. , and Bryan D. , Pregnancy Outcome and Complications in Women With Spina Bifida, Journal of Reproductive Medicine. (2000) 45, no. 9, 743–748. 11027084

[9] Jackson A. B. and Sipski M. L. , Reproductive Issues for Women With Spina Bifida, The Journal of Spinal Cord Medicine. (2016) 28, no. 2, 81–91, 10.1080/10790268.2005.11753803. 15889694

[10] Mishra NandaVV, Aggarwal S. , and Tanvir R. , Successful Pregnancy Outcome in an Operated Case of Lipomeningomyocele: A Rare Case, Journal of Clinical and Diagnostic Research. (2016) 10, no. 9, Qd04–qd5, 10.7860/JCDR/2016/18562.8558, 2-s2.0-84988644291. PMC5072029 27790529

[11] O’Neal M. A. , A Pregnant Woman With Spina Bifida: Need for a Multidisciplinary Labor Plan, Frontiers in Medicine. (2017) 4, 10.3389/fmed.2017.00172, 2-s2.0-85060944409. PMC5645511 29082228

[12] Yang L. and Li C. , Anesthesia Management for Cesarean Section in Pregnant Women With Occult Spina Bifida: A Case Report and Literature Review, International Journal of Surgery Case Reports. (2025) 131, no. C, 10.1016/j.ijscr.2025.111319. PMC12051605 40279995

[13] Todde C. , Aversano M. , Luigi Giuseppe L. M. , Zagari A. , Feole L. , and Frigo M. G. , EP216 Epidural Labour Analgesia Is Not Always Contraindicated in Patients with Spinal Dysraphism: A Tethered Cord Syndrome Case Report, Regional Anesthesia & Pain Medicine. (2023) 48, no. Suppl 1, A158–A158, 10.1136/rapm-2023-ESRA.276.

[14] Tidmarsh M. D. and May A. E. , Epidural Anaesthesia and Neural Tube Defects, International Journal of Obstetric Anesthesia. (1998) 7, no. 2, 111–114, 10.1016/S0959-289X(98)90007-3, 2-s2.0-0031980087. 15321228

[15] Sivarajah K. , Relph S. , Sabaratnam R. , and Bakalis S. , Spina Bifida in Pregnancy: A Review of the Evidence for Preconception, Antenatal, Intrapartum and Postpartum Care, Obstetric Medicine. (2019) 12, no. 1, 14–21, 10.1177/1753495X18769221, 2-s2.0-85047388885. 30891087 PMC6416695

[16] Auger N. , Arbour L. , Schnitzer M. E. , Healy-Profitós J. , Nadeau G. , and Fraser W. D. , Pregnancy Outcomes of Women With Spina Bifida, Disability and Rehabilitation. (2018) 41, no. 12, 1403–1409, 10.1080/09638288.2018.1425920, 2-s2.0-85052881554. 29327608

